# Down regulation of Thrombospondin2 predicts poor prognosis in patients with gastric cancer

**DOI:** 10.1186/1476-4598-13-225

**Published:** 2014-09-28

**Authors:** Ruochuan Sun, Jifeng Wu, Yuanyuan Chen, Mingdian Lu, Shangxin Zhang, Daru Lu, Yongxiang Li

**Affiliations:** The Eighth Department of General Surgery, Hefei, China; Department of Pathology, the First Affiliated Hospital of Anhui Medical University, Hefei, China; State Key Laboratory of Genetic Engineering, Fudan-VARI Genetic Epidemiology Center and MOE Key Laboratory of Contemporary Anthropology, Fudan University, Shanghai, China

**Keywords:** THBS2, Gastric cancer, Angiogenesis, Prognostic biomarker

## Abstract

**Background:**

Thrombospondins (THBSs) are a family of multidomain and secreted matricellular Ca^2+^-binding glycoproteins which has at least five members encoded by independent genes. As a THBSs family member, Thrombospondin2 (THBS2) has been reported to regulate angiogenesis. Nevertheless, the functions and clinical significance of THBS2 still remains unclear in gastric cancer.

**Methods:**

The mRNA and protein expression levels of THBS2 were assessed in 14 paired of gastric cancer specimens and corresponding normal mucosas using quantitative real-time PCR and western blot analysis. Immunohistochemistry of THBS2 and CD34 on population-based tissue microarrays consisting of 129 gastric cancer cases were used to evaluate the prognostic significance of THBS2 and microvessel density (MVD) of each sample. Survival analyses were performed by Kaplan–Meier method and Cox’s proportional hazards model. Colony formation assay, endothelial cell tube formation assay, cell migration assay and apoptosis analysis in MKN-45 and SGC-7901 cell lines were carried out to evaluate the effects of THBS2 on gastric cancer *in vitro*.

**Results:**

85.71% (12 of 14) gastric cancer tissues expressed remarkably lower THBS2 in both mRNA and protein levels than the corresponding normal controls. Consistently, tissue microarray (TMA) results showed THBS2 levels were also inhibited in gastric cancer tissues compared with the normal controls. Moreover, we observed that patients with higher levels of THBS2 were significantly correlated with more favourable prognosis while decreased THBS2 expression were associated with poorer histological grades of gastric cancer. Additionally, our in vitro experiments further demonstrated that overexpression of THBS2 could impede both the proliferation rate and the tube formation of Human umbilical vein endothelial cells (HUVECs) in MKN-45 and SGC-7901 cell lines.

**Conclusion:**

Our study suggests THBS2 is aberrantly expressed in gastric cancer and plays a critical role in cancer progression, which can be a potential prognosis predictor of gastric cancer.

**Electronic supplementary material:**

The online version of this article (doi:10.1186/1476-4598-13-225) contains supplementary material, which is available to authorized users.

## Introduction

Gastric cancer is a highly aggressive and lethal malignancy. A total of 952,000 new stomach cancer cases and 723,000 deaths were estimated to have occurred in 2012, accounting for 6.8% of the total cases and 8.8% of total deaths. Moreover, Both new cases and deaths of gastric cancer in China, ranked 1st globally, accounting for over 40% of that in the world [1]. Therefore great concerns are raised for the researches of gastric cancer.

Thrombospondins (THBSs) are a family of multidomain and matricellular Ca^2+^-binding glycoproteins secreted by stromal fibroblasts, endothelial cells and immune cells [2]. They have at least five members encoded by independent genes. By binding with numerous target proteins, they participate in diverse biologic processes such as angiogenesis, cell motility, apoptosis, cytoskeletal organization, and serve as interaction platforms in the extracellular matrix (ECM) [3, 4]. Notably, THBS1 and THBS2 are special in this family for their type I repeats and both of them are mainly shown as inhibitors of angiogenesis [5, 6], one key part of cancer research [7–9]. Microvessel density (MVD) is a widely used parameter to estimate the degree of angiogenesis in tumours with CD34 being the microvessel maker in gastric cancer [10]. Based on the previous findings [2], we put forward a hypothesis in our study that it could also affect the angiogenesis in gastric cancer.

Besides angiogenesis, THBS2 has been reported to interact with multiple cell receptors, growth factors and ECM proteins as well as regulate apoptosis, cell proliferation and adhesion [11]. In cancer researches, increasing attentions have been focused on THBS2. Tokunaga et al. [12] found that THBS2 expressed in patients with colon cancer exhibited a significant lower risk of hepatic metastases and tumour vascularity compared with the patients whose tumours were THBS2 deficiency. Furthermore, De Fraipont et al. [13] demonstrated that THBS2 was significantly correlated with clinical status and outcome, and for most tumours, there was an inverse correlation between the THBS2 expression level and the degree of their malignancy. In addition, THBS2 also played a key role in breast cancer [14], myeloma [15], malignant melanoma [16], prostate cancer [17] and pulmonary adenocarcinoma [18, 19]. In gastric cancer, other members of THBSs, THBS1 [20] and THBS4 [21] could be as a prognostic biomarker or a powerful marker for diffuse-type gastric adenocarcinomas. However, the detailed function of THBS2 in gastric cancer and its implications for clinical diagnose still remain dismal. Thus, we attempted to unveil the clinical significance of THBS2 and its effect in gastric cancer.

To verify our aforementioned hypothesis, we examined the THBS2 expression levels in human gastric cancer tissues and corresponding normal tissues and then explored the possible correlations between the expression of THBS2 and clinicopathological features, clinical prognosis and the MVD counting in patients with gastric cancer. In addition, colony formation assay, endothelial cell tube formation assay, cell migration assay and apoptosis analysis were carried out in MKN-45 and SGC-7901 cell lines to explore the *in vitro* effect of THBS2 in gastric cancer cells.

## Results

### Patient characteristics and clinical outcomes

In the present study, we enrolled 129 eligible patients, which consisted of 101 males and 28 females. The age of the patients ranged from 29 to 80 years (y) with a median age of 61y. All the clinicopathological characteristics are summarized in Table 1. The median overall survival (OS) of the patients was 57 months, and mean OS was 44.01 [95% confidence interval (CI) 39.78-49.24 months].

**Table 1:** **Association among clinicopathological variables and THBS2 expression and overall survival of gastric cancer patients**

Variables	Negative	Positive	Total No.	***P***value ^a^	Overall survival OS	
					Log-Rank ***P*** ^b^	HR(95% CI)
	No.(%)	No.(%)				
Age (y)				0.112	0.743	
<61	24(38.1)	39(61.9)	63			1(reference)
≥61	35(53.0)	31(47.0)	66			1.09(0.67-1.77)
Gender				0.831	0.405	
Male	47(46.5)	54(53.5)	101			1(reference)
Female	12(42.9)	16(57.1)	28			0.76(0.40-1.45)
Histological grade				**0.005**	**0.011**	
Well and moderate	24(33.8)	47(66.2)	71			1(reference)
Poor and other	35(60.3)	23(39.7)	58			1.89(1.16-3.09)
Tumor location				0.419	0.204	
Upper	27(40.3)	40(59.7)	67			1(reference)
Middle	15(53.6)	13(46.4)	28			1.66(0.86-3.18)
Lower	17(50.0)	17(50.0)	34			1.90(0.90-4.01)
Depth of invasion				0.271	**0.001**	
T1-T2	9(34.6)	17(65.4)	26			1(reference)
T3-T4	50(48.5)	53(51.5)	103			11.60(2.83-47.45)
Lymph node metastasis				0.454	**<0.001**	
Absent	17(40.5)	25(59.5)	42			1(reference)
Present	42(48.3)	45(51.7)	87			5.16(2.46-10.84)
TNM stage				0.283	**<0.001**	
I-II	21(39.6)	32(60.4)	53			1(reference)
III-IV	38(50.0)	38(50.0)	76			7.14(3.52-14.49)

Bold value is statistically significant at *P* < 0.05, HR hazard ratio.

^a^, *P* value of clinicopathological variables and THBS2 expression.

^b^, *P* value of association between clinicopathological variables and overall survival of gastric cancer patients.

### THBS2 is down-regulated in gastric cancer

**Figure 1:**
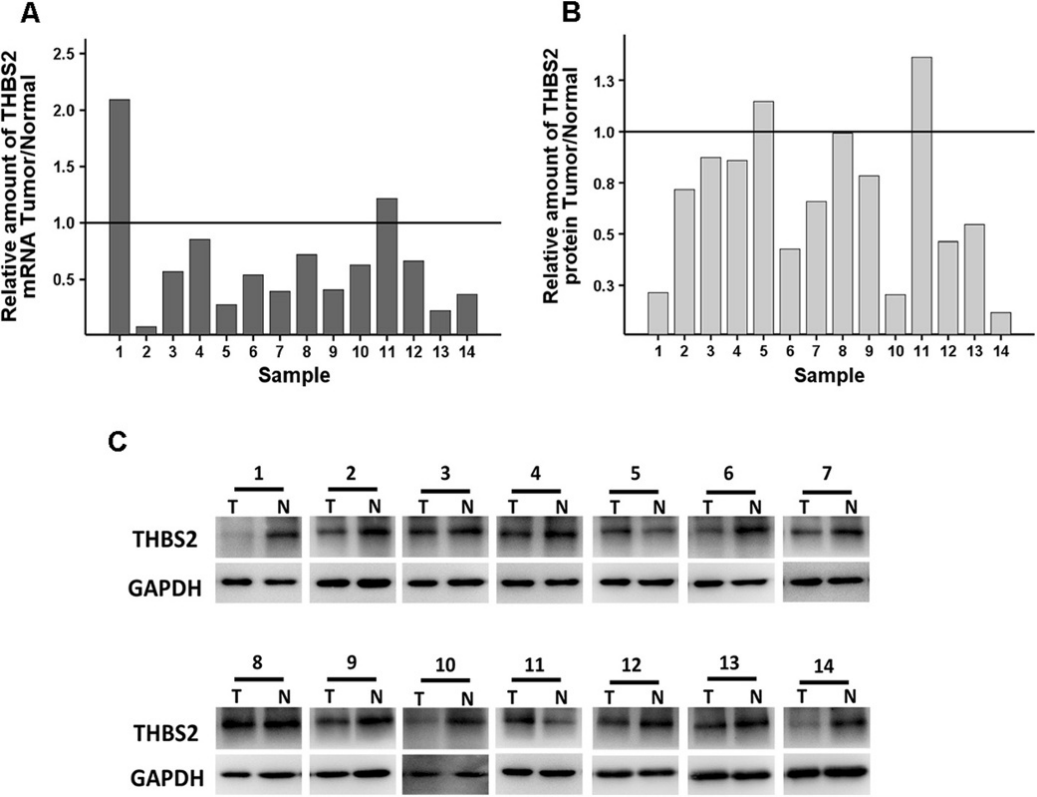
**mRNA and protein expression level of THBS2 in 14 gastric cancer tissues and the corresponding normal tissue. (A)** Relative mRNA expression of THBS2 was detected by quantitative real-time PCR. Expression of normal tissues were normalized to 1.0 as indicated. **(B)** The densitometry analysis results of the THBS2 bands were normalized to GAPDH using Image J. Expression of normal tissues were normalized to 1.0 as indicated. **(C)** Relative protein expression of THBS2 was estimated by Western blot assay.

**Figure 2:**
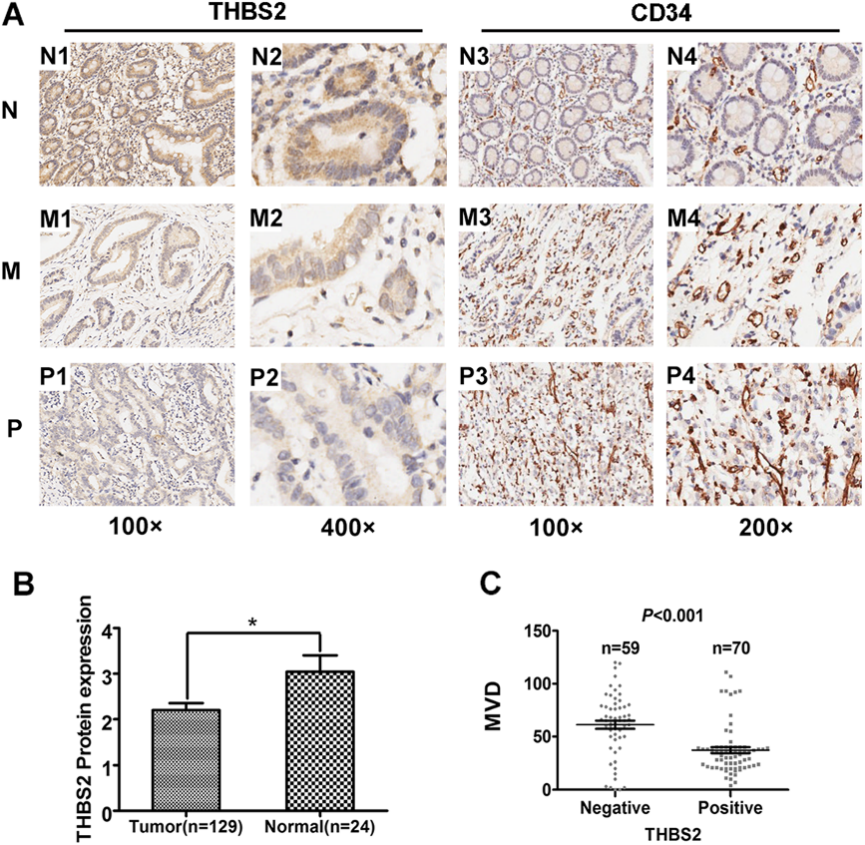
**Immunohistochemical analysis of THBS2, CD34. (A)** Representative images of THBS2 and CD34 expression: N1, N2, N3, N4, normal gastric tissue (N); M1, M2, M3, M4, moderately differentiated (M); P1, P2, P3, P4, poorly differentiated (P). Magnification: 100× (N1, M1, P1, N3, M3, P3), 200× (N4, M4, P4) and 400× (N2, M2, P2). **(B)** Comparison of THBS2 expression of gastric cancer (n = 129) and normal gastric tissues (n = 24) in TMA. The THBS2 expression level presented as mean ± SEM. **(C)** Comparison of MVD in THBS2-positive gastric cancer group (n = 70) and THBS2-negative gastric cancer group (n = 59). Dots represent MVD of each samples. Values are means ± SEM.

In order to determine the expression levels of THBS2 in gastric cancer, we first evaluated the mRNA expression levels of *THBS2* in gastric cancer by quantitative real-time in the second cohort. This cohort of samples, mentioned in Methods, included 14 human gastric cancer and the corresponding normal tissues. As shown in Figure 1A, *THBS2* levels were markedly lower in 85.7% (12 of 14) of the gastric cancer tissues than its normal controls, while the remaining two pairs of the samples exhibited the opposite. Then, we detected the protein expression levels of THBS2 via western blot in the same cohort. As show in Figure1B and C, we observed that THBS2 protein expression levels were decreased in 85.7% (12 of 14) of the tumour samples compared with its corresponding control, while sample 5 and 11 displayed inversely. To confirm this finding, we further performed a tissue microarray (TMA) and assessed the protein expression levels of THBS2 according to the immunoreactivity score (IRS) of each sample. We found THBS2 proteins were mainly expressed in the cytoplasms of gastric cells (Figure 2A), and in accordance with the results of western blot, the levels of THBS2 were significantly inhibited in gastric cancer tissues compared with the normal controls (Figure 2B, P < 0.05). Taken together, our results demonstrates that THBS2 is down-regulated in gastric cancer in both mRNA and protein levels.

### THBS2 expression levels are inverse correlated with histological grades of gastric cancer

To further clarify the clinical importance of THBS2, we analysed the relationship between THBS2 protein expression and clinical features including age, gender, histological grade, tumour location, depth of invasion, lymph node metastasis and TNM stage of gastric cancer patients in THBS2-positive and negative groups. As shown in Table 1, we found a significant inverse correlation with the histological grades of gastric cancer (*P* < 0.01). The histological grades of patients in THBS2-positive group were more likely to be well and moderate, whereas patients in THBS2-negative group tended to be poor and other. However, we failed to discover any significance statistically between the expression levels of THBS2 and other clinical parameters. Collectively, our findings suggest that THBS2 is inverse correlated with histological grade of gastric cancer.

### THBS2 expression levels are inverse correlated with MVD in gastric cancer

Previous reports implied THBS2 could regulate tumour angiogenesis [6], yet none was reported in gastric cancer. Hence, we asked whether there were any correlations between MVD and THBS2 expression levels as well as other clinical parameters in our clinical samples. The MVD in 129 gastric cancer specimens ranged from 0 to 120, with the median of 40 and mean of 48.12 ± 28.99 (mean ± SD) according to the immunoreactivity of CD34 in our TMA. As shown in Figure 2C, THBS2 was significantly correlated with MVD. The MVD in THBS2-negative group was much higher than that in THBS2-positive group (*P* < 0.001). However, we could not observe any statistic differences between MVD and other clinicopathologic features including clinical prognosis in our samples (data not shown). In conclusion, our discoveries demonstrate that THBS2 is inverse correlated with MVD in gastric cancer.

### THBS2 is significantly associated with clinical prognosis of gastric cancer patients

**Figure 3:**
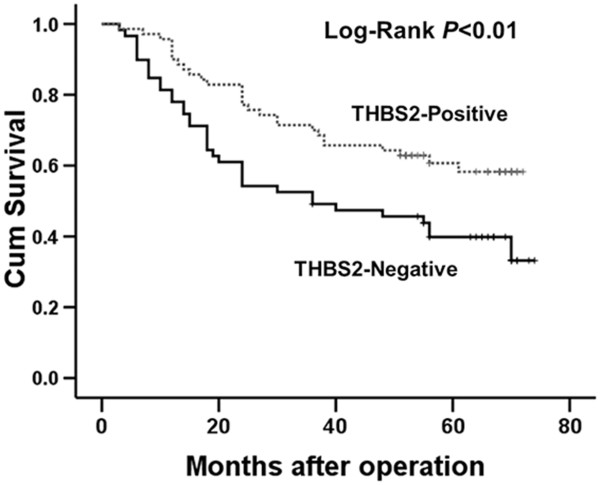
**Kaplan–Meier survival analyses with Log-Rank test for overall survival (OS).** Kaplan-Meier analysis of the correlations between different THBS2 expression levels and OS in 129 gastric cancer patients.

To examine the relationship between THBS2 expression and the clinical prognosis of gastric cancer patients, we conducted survival analyses by univariate and multivariate Cox’s proportional hazards regression model. As shown in Table 1, we found histological grade, depth of invasion, lymph node metastasis and TNM stage were statistically significant factors for OS in univariate analysis. However, since TNM stage also contains the information of depth of invasion and lymph node metastasis, we further performed multivariate survival analyses with adjustment for the histological grade and TNM stage. We noticed that TNM stage (*P* <0.001) and THBS2 expression (*P* <0.01) were both independent prognostic factors for the assessment of patient outcomes. Higher TNM stage was a risk factor for longer OS [*P* <0.001, HR (hazard ratio) = 7.23, 95% CI 3.55-14.69) and elevated expression of THBS2 was yet a favourable factor for longer OS (*P* < 0.01, HR = 0.51, 95% CI 0.31-0.85). Moreover, Kaplan–Meier survival analysis further confirmed that THBS2 expression was significantly correlated with clinical outcomes (Figure 3, P <0.01, n = 129). Patients with lower THBS2 expression displayed a significantly shorter OS [median 36 months, mean 40.07 ± 3.65 (mean ± SE) months], whereas patients with higher THBS2 expression showed a favourable prognosis [median 59 months, mean 53.70 ± 2.95 (mean ± SE) months]. Together, our results demonstrate THBS2 is significantly associated with the clinical prognosis of gastric cancer patients, which might become a prognostic marker for gastric cancer.

### Overexpression of THBS2 inhibits the growth of gastric cancer cells *in vitro*

**Figure 4:**
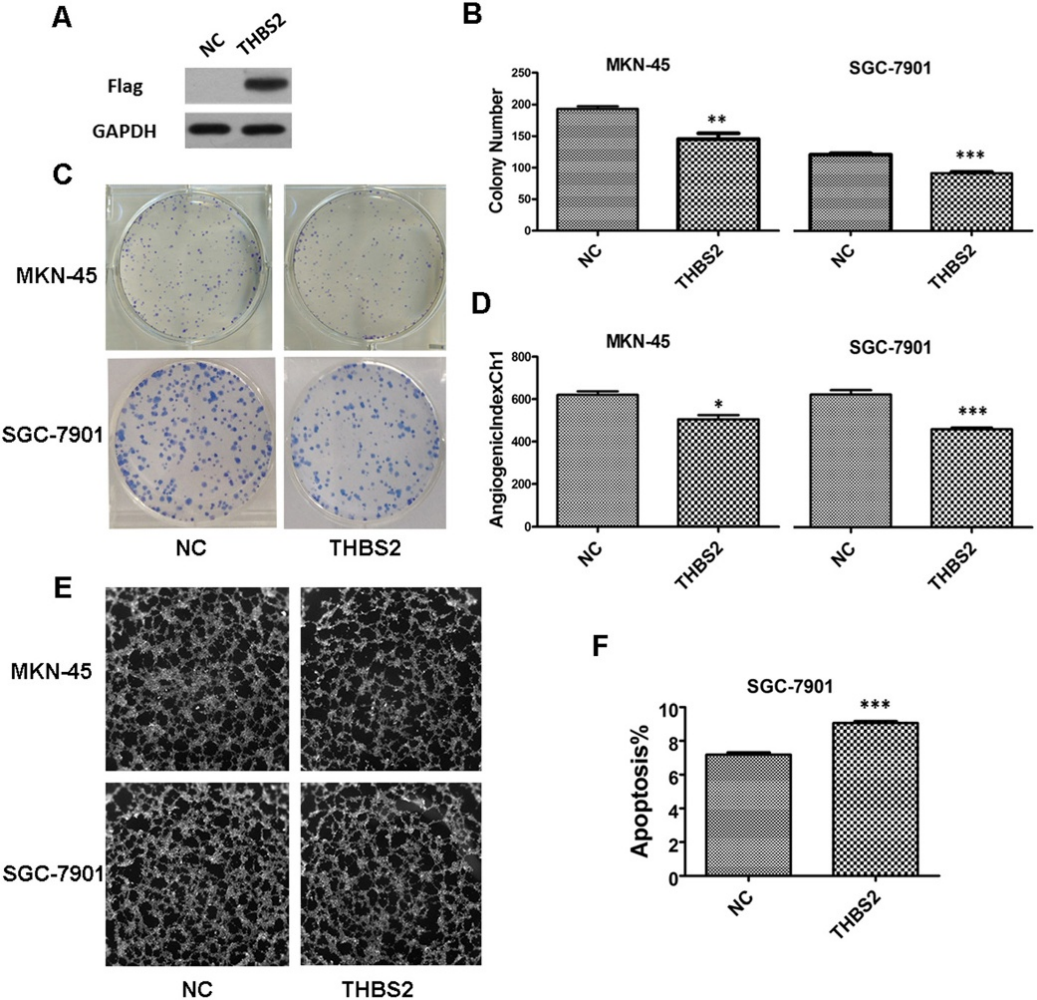
**Overexpression of THBS2 inhibited the proliferation of gastric cancer cells, suppressed the angiogenesis of gastric cancer, and promoted apoptosis in SGC-7901 cell line** ***in vitro*** **. (A)** Western blot analysis of Flag in 293 T cells with THBS2 overexpression. **(B)** Colony number analysis for MKN-45 and SGC-7901 cells with THBS2 overexpression and negative control. Values are means ± SD from three independent experiments. **(C)** Images of colony formation assay. **(D)** AngiogenicIndexCh1 analysis of endothelial cell tube formation assay. Values are means ± SD. **(E)** Images of endothelial cell tube formation assay. **(F)** Analysis of apoptosis in MKN-45 cell line. Values are means ± SD.

Because THBS2 was significantly down-regulated in gastric cancer, we inferred that THBS2 might inhibit growth, promote apoptosis and enhance the migration ability of gastric cancer cells. To verify our hypothesis, we first constructed one lentiviral vector to stable express THBS2 transcripts in gastric cancer cell lines. Altered expression of THBS2 in 293 T cells was confirmed by western blot analysis (Figure 4A). Then, we chose MKN-45 and SGC-7901 cell lines for our *in vitro* study because the mRNA expression levels of THBS2 were lower than others (data not shown). We then performed the colony formation assay to evaluate the effects of THBS2 on the growth of MKN-45 and SGC-7901 cells. As shown in Figure 4B, we found cells with THBS2 overexpression formed significantly fewer colonies on soft agar compared with those of control vector–infected cells (*P* < 0.01 for MKN-45 cells and *P* < 0.001 for SGC-7901 cells, respectively). Moreover, we examined the apoptosis levels between the two groups in both cell lines and discovered that THBS2 promoted apoptosis in SGC-7901 cells (Figure 4F) but not in MKN-45 cells (Additional file 1: Figure S1A). And there were no significant differences between the two groups in SGC-7901 and MKN-45 cell lines in cell migration according to the transwell assays (Additional file 1: Figure S1B and C). In summary, our data support that THBS2 impedes the growth of gastric cancer cells in vitro possibly via the regulation of apoptosis.

### THBS2 suppresses gastric cancer angiogenesis *in vitro*

THBS2 is known as an inhibitor of angiogenesis [2]. To provide evidences of THBS2 regulates the angiogenic phenotype of gastric cancer, endothelial cell tube formation assays were carried out. As shown in Figure 4E and D, in both MKN-45 and SGC-7901 cell lines, human umbilical vein endothelial cells (HUVECs) cultured with the medium from THBS2-overexpression gastric cancer cells showed significantly lower AngiogenicIndexCh1 than the negative control (*P* < 0.05 for MKN-45 cells and *P* < 0.001 for SGC-7901 cells, respectively ). Collectively, our results imply that THBS2 suppresses the angiogenesis of gastric cancer cells *in vitro*.

## Discussion

THBS2 is known as a natural potent inhibitor of angiogenesis and a modulator of the remodelling process [22, 23]. However, none of the former researches discussed the roles of THBS2 and its clinical significance in gastric cancer.

Firstly, we found that mRNA and protein expression levels of THBS2 were down-regulated in most samples. However, one previous research reported THBS2 mRNA expression levels in gastric cancer were higher than normal control [24]. Considering the limited sample size in this study, which incorporated only 6 tissue specimens, it is possible for this difference due to tumour heterogeneity for two pairs of our second cohort did also show the same patterns. Yet our subsequent result of TMA, larger in sample size, was consistent with the result from the second cohort. Taken together, we believed that THBS2 was more likely to be down regulated in gastric cancer cell versus normal mucosa.

Moreover, we also noticed that the relative expression of the samples exhibited differently in mRNA and protein level. Lacking sufficient evidences, we infer that the function of THBS2 within gastric cancer cells may be regulated by some intracellular upstream signalling factors, protein ubiquitination, and other possible manners, which may affect the protein levels of THBS2 and the phenotypes in gastric cancer cells. And further emphasis can be put on this interesting phenomenon. Nevertheless, the majority of the samples from our second cohort displayed the same expression patterns between mRNA and protein levels.

Secondly, our data showed that THBS2 could promote apoptosis only in SGC-7901 cells but not in MKN-45 cells. Former researches showed that an N-terminal recombinant fragment of THBS2 could activate CD36-mediated endothelial cell apoptosis [14], and the combination of CD36 and THBS2 could activate the caspase signalling pathway [25]. Thus, we assumed that the heterogeneity of the two cell lines used in our study, like differentiated CD36 expressions, might partly be the reason of different levels of apoptosis induced by THBS2, which called for further investigations.

Thirdly, our results showed that THBS2 could suppress angiogenesis of gastric cancer. It is known that tumour proliferation, invasion, or metastasis are dependent on angiogenesis [26]. And based evidences support our discovery and point out that the possible mechanisms that THBS2 inhibits angiogenesis: 1) an apoptosis-independent fashion by inducing endothelial cell apoptosis, cell cycle arresting and decreasing of endothelial cell migration [27], 2) dependent on caspase signalling pathway activated by CD36 [28]. 3) functioning as co-receptors for the low density lipoprotein receptor related protein (LRP1), which could clear the complexes of THBS2 with matrix metalloproteinases-2, -9 or vascular endothelial growth factor (VEGF) from the pericellular environment of mesenchymal cells [29–32].

Lastly, MVD is one of the most commonly used parameters to assess the degree of tumour angiogenesis and previous studies have found MVD could be an independent prognostic factor in gastric cancer and other tumour [33–35]. In our study, we found MVD was significantly correlated with THBS2 protein expression (*P* < 0.001) in gastric cancer, but not with clinical features and clinical prognosis. These results demonstrated that THBS2 could affect angiogenesis in gastric cancer, nevertheless, because of no correlation between MVD and prognosis in our samples, we suspected that higher THBS2 expression indicating longer survival was not merely through inhibiting angiogenesis of gastric cancer, other mechanisms such as regulating ECM remoulding [23] and proliferation rate of gastric cancer cells might also be the reasons that need further researches to explore.

Collectively, our findings emphasized on the important roles of THBS2 in prognostic significances as well as tumour proliferation and angiogenesis in gastric cancer. These data suggest THBS2 could be an important prognostic marker for gastric cancer patients. Moreover, based on the effects of THBS2 on tumour development and angiogenesis, our researches provide valuable clues for clinical practices to develop molecular inhibiting therapeutics using targets deduced from the biological knowledge provided by the THBS2 signature.

## Conclusion

Our study presents the first line of evidences that THBS2 expression is down-regulated at both mRNA and protein levels and decreased THBS2 expression is associated with the poor histological grade of gastric cancer. Crucially, overexpression of THBS2 has a significant correlation with favourable prognosis of gastric cancer patients. On the other hand, THBS2 affected prognosis in gastric cancer may not only through regulating the angiogenesis, but in some other manners like regulating ECM remoulding and inhibiting the proliferation of gastric cancer cells. These findings suggest that THBS2 could be a potentially critical role of in the pathogenesis and progression of gastric cancer. We confirm that, with the gradual depth studies of THBS2, it may be a promising useful and simple biomarker for predicting clinical outcome for gastric cancer patients.

## Methods

### Patients and tissue specimens

In the present study, we incorporated two cohorts of specimens. The first cohort contained 129 formalin-fixed, paraffin-embedded human gastric cancer and 24 randomly selected normal gastric tissues. They were consecutively recruited between December 2006 and May 2008 from Department of General Surgery, The First Affiliated Hospital of Anhui Medical University, Hefei, China. These specimens were incorporated into TMA for immunohistochemical staining. Histological features of these specimens were confirmed by pathologists. Pathological TNM staging was evaluated according to the 2010 criteria of The American Joint Committee on Cancer (AJCC). The second cohort was composed of 14 gastric cancer patients who undergone tumour resection therapy at the Eighth Department of General Surgery, The First Affiliated Hospital of Anhui Medical University. The fresh gastric cancer tissue and corresponding normal mucosa (at least 5 cm distant from the tumour edge) were immediately frozen in liquid nitrogen and stored at -80°C until using for Quantitative real-time PCR and Western blot analysis. Patients in both cohorts did not receive preoperative chemotherapy and/or radiation. Written informed consent was provided by all participants. All specimens were handled anonymously according to the ethical standards. This study was conducted with the approval of the Ethical Review Committee of the hospital.

### RNA extraction and quantitative real-time PCR

Total RNA was extracted from the second cohort using Trizol reagent (Invitrogen) according to the manufacturer’s protocol. cDNA was synthesized by random primers and Superscript II reverse transcriptase (Toyobo, Osaka, Japan). The primers used for amplification for THBS2: forward primer 5’-CGTGGACAATGACCTTGTTG-3’ and reverse primer 5’-GCCATCGTTGTCATCATCAG-3’. Glyceraldehyde-3-phosphate dehydrogenase (GAPDH) was amplified in the same q-PCR as an internal control using primers: forward primer 5’-AGCCACATCGCTCAGACAC-3’ and reverse primer 5’-GCCCAATACGACCAAATCC-3’. The reaction ran on the ABI 7900HT Sequence Detection System (Applied Biosystems, CA, USA) in the presence of SYBR-Green dye (Toyobo, Osaka, Japan). The reaction condition was a denaturation program (95°C for 5 min), and an amplification and quantification program for 40 cycles (95°C for 15 s and 60°C for 45 s). Every sample was tested in triplicates, and a melting curve analysis of each sample was used to check the specificity of amplification. The expression level was determined as a ratio between THBS2 and the internal control GAPDH in the amounts of mRNA calculated by comparative CT method.

### Protein extraction and Western blot

Total protein were extracted from 14 frozen gastric cancer and its corresponding normal mucosa tissue by ice in radio immunoprecipitation assay Lysis Buffer (RIPA; Beyotime institute of Biotechnology, Jiangsu, China), and measured using a BCA protein assay kit (Beyotime institute of Biotechnology, Jiangsu, China). The lysate was centrifuged at 12000 rpm for 5 minutes, and the supernatant was heated at 100°C for 5 minutes. Then equivalent proteins of each pair specimens were separated on 8% sodium dodecyl sulfate polyacrylamide gel electrophoresis and electrotransferred to polyvinylidene fluoride membranes (Millipore, Billerica, MA). After The blocked with TBST containing 5% skim milk in at room temperature for one hour ,the membranes were blotted with anti-THBS2 antibody (1:1500; Novus) and anti-Flag (1:3000; Sigma). Followed membranes incubation with horseradish peroxidase–labelled anti-rabbit and anti-mouse IgG as the secondary antibody (Beyotime institute of Biotechnology). Anti-GAPDH antibody (1:4000; Aogma) was used as a loading control antibody. The bound antibodies were detected with the enhanced chemiluminescence method.

### Immunohistochemistry and TMA analysis

Gastric cancer and normal gastric mucosa specimens were formalin-fixed, paraffin-embedded and were used to construct a tissue TMA. H&E-stained slides were screened to identify optimal intratumoural tissue for analysis. 4-μm thick sections were baked at 60°C for 1 hour, deparaffinised with xylenes, and rehydrated in graded ethanol to distilled water. Antigen retrieval was achieved with placing the sections with citrate buffer in a rice steamer for 30 minutes. To quench the endogenous peroxidase activity, the sections were treated with 3% H_2_O_2_ in methanol. Then we used 1% bovine serum albumin to block the nonspecific binding. Anti-THBS2 antibody (Novus, 1:2000) and anti-CD34 (Abcam, 1:250) were incubated with the sections at 37°C overnight, then incubated with horseradish peroxidase-labelled Anti-rabbit IgG as the secondary antibody (Life Technologies). In the end, slides were placed on an autostainer link instrument and proceed with staining. For negative controls, the primary antibodies were replaced with normal rabbit serum. The final effective immunohistochemical staining was evaluated by two independent pathologists without knowing the information of patients according to the staining intensity and extent of staining. Tissue section was scored as the percentage of stained cytoplasm in gastric cancer gland cells and normal gland cells (0 points for no cells stained, 1 point for <25%, 2 points for 25-75%, and 3 points for >75% of cells stained), and the staining intensity of immunoreactivity was graded on a scale of 0 to 3. The immunoreactivity score (IRS) was resulted from the multiplication of both parameters. Specimens were scored as follows: negative (IRS = 0 ~ 2), positive (IRS = 3 ~ 9).

### MVD counting

Immunohistochemical staining of CD34 and generally accepted criteria performed by Weidner et al. [36] were used for MVD counting. Any stained endothelial cells or endothelial cell clusters were separated from adjacent microvessels, and the thickness of every vessel wall over 2.75 μm would be excluded. Three separate sections containing hot-spots (where the highest number of discrete microvessels was stained) in each sample of TMA were chosen in low magnifications (100×) under a light microscope (Leica, Germany). Subsequently, microvessels were counted in each section in high magnification (200×). Final counts were expressed as the average of all the three sections examined.

### Cell culture and lentivirus infection

Gastric adenocarcinoma cell line MKN-45 and SGC-7901 were obtained from the Shanghai Institutes for Biological Sciences, Chinese Academy of Sciences. These two cell lines were grown in RPMI1640 medium (Gibco, USA) supplemented with 10% fetal bovine serum (FBS, Gibco, USA), and incubated at 37°C in a humidified atmosphere with 5% CO_2_. The maintenance of MKN-45 and SGC-7901 cell lines were described previously [37]. Human 293 T cells were maintained in DMEM with 10% fetal bovine serum (Gibco, USA). To generate cell lines overexpressing THBS2, the open reading frame of THBS2 [GenBank:NM_003247.3] was cloned into the lentiviral vector GV287 (Ubi-MCS-3FLAG-SV40-EGFP; GeneChem Co., Ltd), and GV287 empty vector served as negative control (NC) for Ubi-THBS2-3FLAG-SV40-EGFP. The procedures of packaging and infection of lentivirus were according to the previous study [38].

### Colony formation assay

Colony formation was performed as described previously [39, 40]. 8 × 10^2^ cells suspended in DMEM medium containing 10% fetal bovine serum were plated in 6-well plates. The plates were incubated at 37°C in a 5% CO_2_ incubator for 14 days or colonies with more than 50 cells were counted. Each dose was done in triplicate, and the experiments were done at least three times.

### Endothelial cell tube formation assay

The tube formation assay was done as described previously [41]. 70 μL of Matrigel Basement Membrane Matrix (BD Biosciences) were pipetted into each well of a 96-well plate and polymerized for 30 minutes at 37°C. HUVECs were harvested after trypsin treatment and suspended in conditioned medium from THBS2 overexpression group and NC group. Then 2 × 10^4^ HUVECs with these conditioned medium were added to each well and incubated at 37°C, 5% CO_2_, for 20 hours. The cultures were stained with Cellomics Cytoskeletal Rearrangement Kits (Thermo Fisher Scientific) according to the protocol by the company and analysed with Cellomics (Thermo Fisher Scientific). The measurement is AngiogenicIndexCh1 which is defined as 1000 × Total Area of Connected Tubes/Total Image Area(Thermo Fisher Scientific provided).

### Cell migration assay

Cell migration assays were examined according to previous study [42]. 1 × 10^5^ cells in 100 μL DMEM medium without FBS were seeded on a fibronectin coated polycarbonate membrane insert in a Transwell apparatus (Corning, NY, USA). 600 μl DMEM containing 10% FBS was added into the lower chamber. When the 20 hours incubation was completed, cells were fixed with methanol and stained with Giemsa. Cell numbers on the lower side of the filter were counted under a light microscopy (Olympus micropublisher 3.3RTV). Each dose was done in triplicate, and the experiments were done at least three times.

### Apoptosis analysis

Apoptosis analysis was described in detail previously [37]. These assays used apoptosis kit (eBioscince, USA ) following to the manufacturer’s instructions. The stained cells were analysed by flow cytometry (FACSCalibur, BD).

### Statistical analysis

SPSS 15.0 software (SPSS, Inc. Chicago) was used for all statistical analyses. Fisher’s exact test or The Pearson χ^2^ test was used to analyse the relationship between THBS2 protein expression level and the clinicopathologic features. Mann-Whitney U test used for comparing MVD with THBS2 expression and clinicopathologic features as well as the protein expression level between gastric cancer tissue and normal tissue in the TMA. Survival curves calculation and OS curve plotting used the Kaplan–Meier method, and the Log-Rank test was applied to compare the distribution between groups. Multivariate Cox’s proportional hazards models were used to explore the effects of the clinicopathologic variables with THBS2 protein expression level on survival. Comparisons of quantitative data were analyzed by Student’s t-test between two groups (two-tailed). *, **, or *** indicates *P* < 0.05, *P* < 0.01, or *P* <0.001, respectively. *P* < 0.05 was defined as statistically significant.

## Electronic supplementary material

Additional file 1: Figure S1: Apoptosis analysis of MKN-45 cell line and cell migration assay. (A) Analysis of apoptosis in MKN-45 cell line. Values are means ± SD. (B) Migration number counted and analysis under 400× microscope. (C) Images of colony migration assay (400×). (TIFF 4 MB)
