# Supplementary figures and images for: Down regulation of Thrombospondin2 predicts poor prognosis in patients with gastric cancer
Source: Mol Cancer. 2014 Sep 28;13:225. doi: 10.1186/1476-4598-13-225 (PMC4189190; doi:10.1186/1476-4598-13-225)

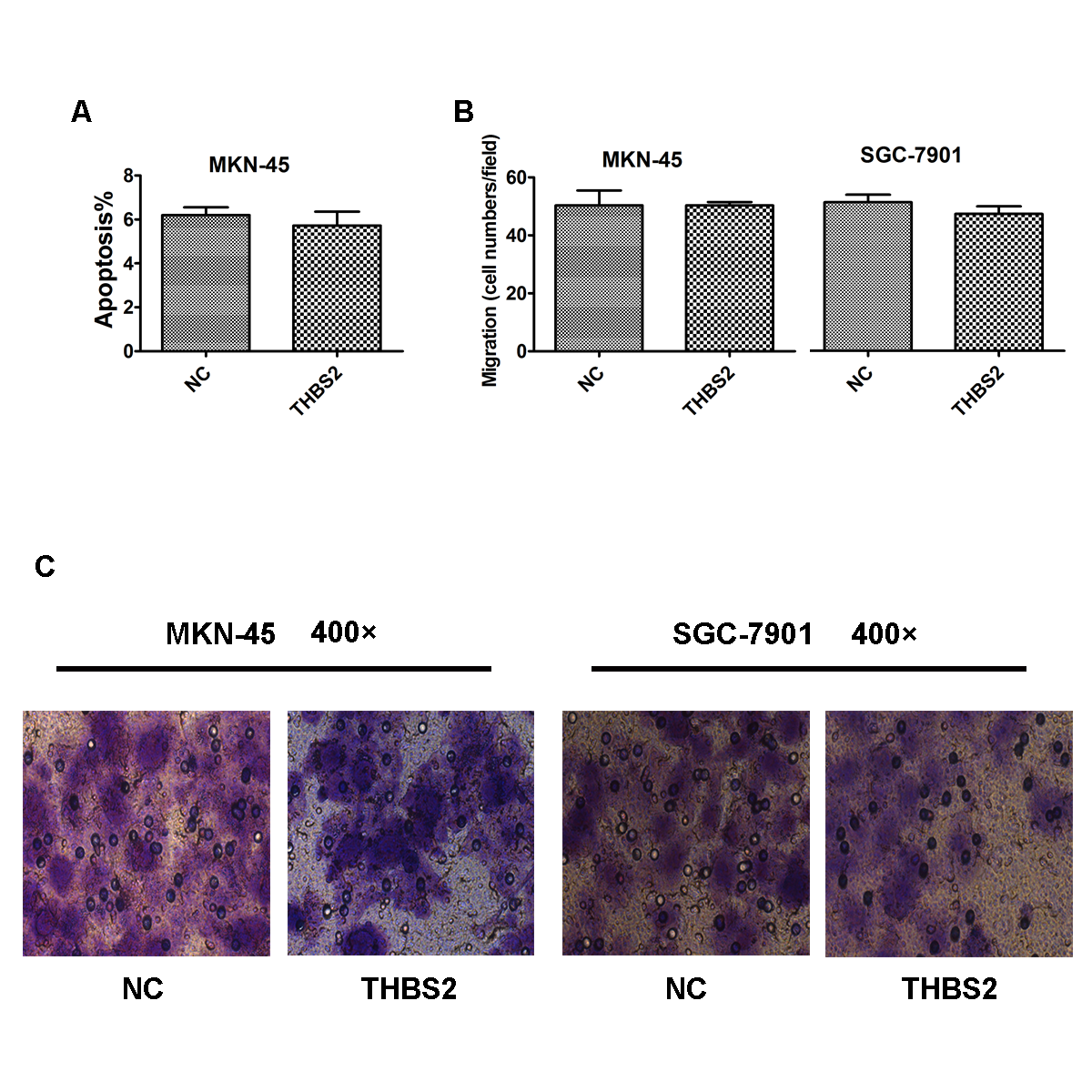

Supplement: Supplementary file 1 — Additional file 1: Figure S1: Apoptosis analysis of MKN-45 cell line and cell migration assay. (A) Analysis of apoptosis in MKN-45 cell line. Values are means ± SD. (B) Migration number counted and analysis under 400× microscope. (C) Images of colony migration assay (400×). (TIFF 4 MB) [file 12943_2013_1423_MOESM1_ESM.tiff]
